# Non-compliance to the Tobacco Control and Regulatory Act among Vendors in the Vicinities of Schools of a Metropolitan City: A Descriptive Cross-sectional Study

**DOI:** 10.31729/jnma.7206

**Published:** 2022-10-31

**Authors:** Ankush Kumar Gupta, Dip Narayan Thakur, Mukesh Kumar Sah, Rakesh Yadav, Prem Lal Basel

**Affiliations:** 1 Maharajgunj Medical Campus, Maharajgunj, Kathmandu, Nepal; 2 Nepal Public Health Foundation, Maharajgunj, Kathmandu, Nepal; 3 Central Department of Public Health, Institute of Medicine, Maharajgunj, Kathmandu, Nepal

**Keywords:** *government regulations*, *primary schools*, *vendors*

## Abstract

**Introduction:**

Tobacco use is the underlying cause of ill health, preventable deaths, and disabilities worldwide. The Tobacco Product Control and Regulation Act 2011 prohibits the sale of tobacco in public places including educational institutions but non-compliance to the law had not been assessed. This study aimed to find out the prevalence of non-compliance to the Tobacco Product Control and Regulation Act among vendors in the vicinities of schools in a metropolitan city.

**Methods:**

This descriptive cross-sectional study was conducted in a metropolitan city in August 2018. Ethical approval was taken from Institutional Review Committee [Reference number: 23(6-11-E)2/075/076]. A convenience sampling method was used to recruit vendors within 100 meters radius of secondary schools. The data were collected through face-to-face interviews using a semistructured questionnaire. Point estimate and 95% Confidence Interval were calculated.

**Results:**

Out of total 217 vendors, non-compliance to the section 3 of section 11 of Tobacco Product Control and Regulation Act was found in 195 (89.86%) (85.84-93.88 at 95% Confidence Interval). Among the non-compliers, 110 (56.41%) were selling both smoked and smokeless tobacco products, 78 (40%) were selling smoked and 7 (3.59%) were selling smokeless tobacco products.

**Conclusions:**

The non-compliance with Tobacco Product Control and Regulation Act's prohibition of tobacco sales within 100 m of schools in Kathmandu Metropolitan was similar with other studies conducted in similar settings.

## INTRODUCTION

Tobacco use is a significant contributor to ill health, preventable death and disability worldwide.^1^ The tobacco epidemic is attributed to more than eight million annual deaths globally.^2^ Nepal ratified the Framework Convention on Tobacco Control (FCTC) in November 2006. Guided by FCTC, Tobacco Product Control and Regulation Act (TPCRA) came into force in May 2011. Subsection 3 of section 11 of the TPCRA 2011 prohibits the sale of tobacco within 100 meters of public places, including all educational institutions.^3^

The tobacco consumption in general population of Nepal is high (28%) with average age of initiation of 17.8 years.^4^ Monitoring of tobacco use and prevention policies on a regular basis is essential to reverse tobacco epidemic.^5^

This study aimed to find out the prevalence of noncompliance to the Tobacco Product Control and Regulation Act among vendors in the vicinities of schools in a metropolitan city.

## METHODS

A descriptive cross-sectional study was conducted among the vendors having shops within a 100 m radius of secondary schools of the Kathmandu Metropolitan City of Nepal. Data were collected in August 2018. The ethical approval was taken from Institutional Review Committee of Institute of Medicine, Tribhuvan University, Kathmandu, Nepal [Reference number: 23(6-11-E)2/075/076]. Convenience sampling method was used. We selected a total of seventeen schools which included 6 government and eleven private secondary schools. The vendors who provided a consent were included in the study. Those who didnot provide a consent were excluded from the study. The sample size was calculated using the formula,


$$\begin{array}{ll} \text{n} & ={\text{Z}}^{2}\times \frac{\text{p}\times \text{q}}{{\text{e}}^{\text{2}}}\\ & =1.96^{2}\times \frac{0.50\times 0.50}{0.07^{2}}\\ & =196 \end{array}$$

Where,

n= minimum required sample size
Z= 1.96 at 95% Confidence Interval (CI)
p= prevalence taken as 50% for maximum sample size calculation
q= 1-p
e= margin of error, 7%

A minimum required sample size of 196 was obtained. However, a total of 217 samples were included into the study.

The data was collected face-to-face using a semistructured questionnaire with vendors. The items of the tool were guided by TPCRA 2011,^3^ Tobacco Products (Control and Regulatory) Regulation 2012^6^ and Nepal Demographic and Health Survey 2016.^7^ The tool consists of items related to non-compliance, sociodemographic factors, outlet types and inspection by the authority. The non-compliance was assured when the vendor was not following subsection 3 of section 11 of TPCRA and on observation found packed or unpacked tobacco products inside or outside of the shop.

The responses were checked on daily basis and cleaned it before entering the data into Epidata version 3.0. Data were analyzed using IBM SPSS Statistics trial version. Point estimate and 95% CI were calculated.

## RESULTS

Out of total 217 vendors, non-compliance to the section 3 of section 11 of TPCRA was 195 (89.86%) (85.84-93.88, 95% CI). Among the non-compliers, 110 (56.41%) were selling both smoked and smokeless tobacco products, 78 (40%) were selling smoked and 7 (3.59%) were selling smokeless tobacco products (Table 1).

**Table 1:** Type of products sold by non-compliers (n= 195).

Type of product	n (%)
Smoked	78 (40)
Smokeless	7 (3.59)
Both	110 (56.41)

**Figure 1:**
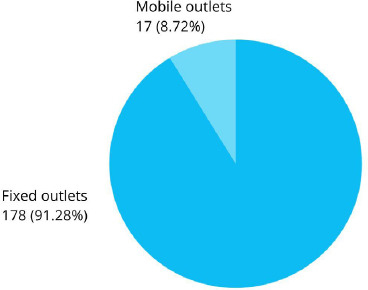
Types of outlets among non-compliers within 100 m radius of schools (n= 195).

One hundred seventy eight (91.28%) retails outlets were predominantly fixed type which comprised of grocery shop, bakery, tea stall, paan (betel nut) shop and supermarket compared to mobile shop such as ghumti pasal (moving shop) and thela (handcart) (Figure 1).

The mean age of the non-compliers was 36.11±8.78 years. Out of total non-compliers, there were 121 (62.05%) males while remaining 74 (37.95%) were females. Most of the vendors were literate 181 (92.82%). Sixty four (32.82%) vendors were consuming tobacco products. Among tobacco consumers, 35 (54.68%) were consuming smoked product, 10 (15.62%) were consuming smokeless products, and 19 (27.70%) were consuming both products (Table 2).

**Table 2:** Socio-demographic characteristic of non­complying vendors (n= 195).

Characteristics	n (%)
**Sex**	
Male	121 (62.05)
Female	74 (37.95)
**Educational status**	
Secondary level and above	82 (42.05)
Below secondary level	99 (50.77)
Illiterate	14 (7.18)
**Vendor currently consuming tobacco product**	
Consuming	64 (32.82)
Not consuming	131 (67.18)
**Types of tobacco product currently consuming (n= 64)**	
Smokeless tobacco product	10 (15.62)
Smoked tobacco product	35 (54.69)
Both products	19 (29.69)

Among the non-compliers, only 47 (24.10%) of the vendors had heard about the TPCRA 2011, out of which 40 (85.10%) of participants specifically knew that prohibition of tobacco sales within 100 meters of the educational institutions. Thirty four (17.43%) shopkeepers reported inspection by authorities to monitor the compliance with the TPCRA. Among 34 (17.43%) inspected vendors, the inland revenue department in 9 (26.48%) vendors. Similarly, 2 (1.02%) vendors reported being fined for violating the TPCRA (Table 3).

**Table 3:** Vendors' knowledge on few sections of TPCRA and inspection practice by various authorities (n= 195)

Characteristics	n (%)
**Heard about TPCRA 2011**	
Yes	47 (24.10)
No	148 (75.90)
**Heard about prohibition within 100 m (n= 47)**	
Yes	40 (85.10)
No	7 (14.90)
**Inspection by authority**	
Yes	34 (17.43)
No	161 (82.57)
**Types of authority (n= 34)**	
Inland Revenue Department	9 (26.48)
Ministry of Forest and Environment	5 (14.70)
Local Police	5 (14.70)
Kathmandu Metropolitan Office	4 (11.76)
Department of Food technology and Quality Control and Drug Law Enforcement Unit, Home Ministry	3 (8.83)
Unknown authorities	8 (23.53)
**Have you ever been fined**	
Yes	2 (1.02)
No	193 (98.98)

Only 4 (2.05%) outlets had signage board indicating tobacco is not sold there, however they found were selling. Prevalence of tobacco sales by minor and pregnant were 11 (5.64%) and 15 (7.70%) respectively. The prevalence of tobacco sales to minors and pregnant women were 122 (61.3%) and 98 (49.8%) respectively (Table 4).

**Table 4:** Conformance to the other tobacco laws among non-compliers (n= 195).

Characteristics	Yes n (%)	No n (%)
Presence of signage board indicating tobacco is not sold	4 (2.05)	191 (97.95)
Size of signage board 20x30 cm	-	195 (100)
License to sell tobacco	0 (0)	195 (100)
Advertisement of tobacco products	5 (2.56)	190 (97.44)
Attractive display to sell tobacco products	4 (2.05)	191 (97.95)
Tobacco sold by minors (under 18 years)	11 (5.64)	184 (94.36)
Tobacco sold by pregnant	15 (7.70)	180 (92.30)
Tobacco sold to minors (under 18 years)	122 (62.56)	73 (37.44)
Tobacco sold to pregnant	98 (50.25)	97 (49.75)
Note: Above laws were extracted from, Nepal's TPCRA 2011, The Tobacco Products (Control and Regulatory) Regulation 2012 and The Tobacco Products (Control and Regulatory) Directives 2014.		

## DISCUSSION

The findings of this study talk about the noncompliance status of TPCRA and its associated factor. In this study out of 217, most 195 (89.86%) vendors were non-complying with subsection 3 of section 11. Similar studies conducted in Delhi and Mangalauru cities of India reported high non-compliance 84.22% (n=57) and 95.84% (n=48) with comparable regulations prohibiting tobacco sale by vendors near schools.^8,9^ Similarly qualitative study in Nepal found that the tobacco policy and laws focused on control of tobacco on use among youth were not implemented properly.^10^ A contrasting findings from Mohali district of Punjab state of India showed non-compliance rate of 7.7% only.^11^ The variability in findings could be due to enforcement and socio-religious factors.

In Mumbai the tobacco selling outlets are mostly permanent (67.6%) almost same as shown by our finding in Kathmandu (91.28%). The display of signage board prohibiting sale of tobacco products in Mumbai is 49%, in Delhi is 38% , in Mangaluru is 10% and in our study it is only 4%.^8,9,12^ This indicates that the awareness level of signage board is less in Kathmandu.

Self-tobacco consumption was 32.8% in our study while study in Mumbai also reported similar stat of 53.2%. Mumbai people consume more smokeless form to tobacco (77%) compared to Kathmandu citizens who are consuming mostly smoked tobacco products (54.7%).^12^ Studies showed that easy access to tobacco among students and adolescent is one of reason for increased consumption as well as sale in Asian countries including Nepal.^10,13^

Active enforcement is necessary for the policy implementation.^14^ Poor enforcement and lack of awareness by law enforcement agents have hindered implementation of tobacco control policies in Kenya and Uganda.^15,16^ Enacting the law without robust measures for enforcement has led to widespread non-compliance for tobacco control legislation in the metropolitan city of Mumbai.^12^ Proactive participation of schools and legal systems has been recommended to enforce and decreases children's tobacco exposure.^9^

Closing all the factories and industries would stop the production and hence sales of tobacco products. The potential of tobacco taxation to raise revenue cannot be ignored. In China conservative estimates suggest that 10% increase in tobacco taxation there is increase in revenue by 5%.^16^ Also, the economy of Nepal is dependent on tobacco business and economic scholars supports for increased tobacco taxation.^17^

This study has some limitations. Study cannot be generalized as the sampling method was nonrandom. The study also possesses respondent bias. The vendors may be unwilling to report that they are violating the law due to fear of legal sanctions. The study was done only in metropolitan city so it may not reflect TPCRA enforcement near secondary schools of rural areas. The data was only collected in between 10 am to 6 pm on working days.

## CONCLUSIONS

The non-compliance with TPCRA's prohibition of tobacco sales within 100 m of schools in Kathmandu Metropolitan City was similar with other international studies. As such, we recommend stricter government monitoring of tobacco sales around schools with special focus on students and teachers, active enforcement and regular inspection of the law and providing alternative business opportunities and increase sustainable livelihood measures for vendors to reduce the prevalence.
